# Secondhand smoke and breast health awareness among Vietnamese American families in California: A mixed-methods study

**DOI:** 10.18332/tpc/224522

**Published:** 2026-08-28

**Authors:** Jimi Huh, Vivian H. Tran, Artur Galimov, Ashley N. Truong, Rachel C. Ceasar, Lawrence A. Palinkas, Thu Tran

**Affiliations:** 1 Department of Population and Public Health SciencesKeck School of Medicine, University of Southern CaliforniaLos AngelesCaliforniaUnited States; 2 Herbert Wertheim School of Public Health and Human Longevity Science, University of CaliforniaSan DiegoCaliforniaUnited States; 3 BPSOS-Center for Community AdvancementWestminsterCaliforniaUnited States

**Keywords:** Vietnamese Americans, breast cancer, secondhand smoke, mixed methods, community-partnered research

## Abstract

**Introduction:**

Tobacco exposure is a preventable breast cancer risk factor. High smoking rates among Vietnamese American (VA) men place VA women at elevated secondhand smoke exposure (SHSe) risk; yet research on the extent to which VA women are aware of SHSe and breast cancer risk is limited.

**Methods:**

This community-partnered, mixed-methods, cross-sectional study examined SHSe prevalences, knowledge, attitudes, and beliefs about SHSe as a breast cancer risk factor among VA women (aged 18–65 years) and explored multilevel factors contributing to SHSe within VA families in Southern California, December 2024 – January 2026. We recruited self-identifying VA women (n=179) via a community-based, snowball strategy for a 30 min survey, and then conducted 60 min one-on-one interviews with 22 VA individuals (11 family dyads).

**Results:**

Current SHSe prevalence was 43% (vs 25.2% nationally); 52.0% reported indoor SHSe during childhood. Only 63.7% had explicit home smoking rules, despite 99.4% believing SHSe affects children’s health and 85.2% linking SHSe to increased breast cancer risk. A Rapid Group Analysis Process of the interview data revealed 3 themes, corroborating survey findings: 1) Religious/spiritual influences led participants to attribute health outcomes to fate or divine will, undermining preventive behaviors and beliefs, 2) A lack of available health information contributed to uncertain/diverging views on breast cancer causes; and 3) Non-smoker women expressing challenges navigating familial smoking-related tension while maintaining relational harmony.

**Conclusions:**

Our findings highlight the importance of family-centered communication while accounting for gender-related tension and family roles to promote cessation, breast cancer prevention and smoke-free spaces.

## Introduction

Breast cancer is the most diagnosed cancer in American women^1^, with racial/ethnic disparities in late-stage presentation, suboptimal treatments and mortality risks^2^. Vietnamese American (VA) women are predicted to have greater increase in breast cancer incidence at 2.1% annual increase (vs 0.5% White, 0.7% Black women) and exhibit higher rates of human epidermal growth factor receptor 2 subtypes^3^, with a lower screening rate (64% vs national average 76%)^4^.

Numerous studies have shown associations between tobacco exposure and elevated breast cancer risk^5-7^. A meta-analysis^8^ reported increased breast cancer risk associated with active smoking (OR=1.08–1.13) and with passive smoking (OR=1.07–1.30). Recent literature on biomarkers derived from carcinogens has shown that secondhand smoke exposure (SHSe) can result in epigenetic modifications, including changes in DNA methylation^9^. Furthermore, studies have shown that SHSe through premenarche up to the first childbirth has greater impact compared to later-life exposure^10,11^. Subgroup racial/ethnic variations in breast cancer risk and related risk factors add further complexity for women who have immigrated at different points of their lives and have experienced differential exposure to SHSe across their lifespan.

The prevalence of smoking among VA men is higher than all Asian American and US men in general (25–37% vs 20.1% and 17.3%, respectively). The smoking rates are higher among VA men with limited English-proficiency, reaching up to 45% – one of the highest in the US^12,13^. These disparities place VA women at heightened risk for SHSe, despite low active smoking rates (2.8–7.9%)^14,15^. One past study documented that ~45% of VA women experience SHSe at home, and 42.4% are exposed in indoor workplaces^16^, relative to the national average of 24.3%^17^. However, a gap remains in research on SHSe in relation to breast cancer risk among VA women, 60% of whom are immigrants^18^.

Data on the knowledge, attitude, and behaviors (KAB) regarding breast cancer and related risk factors in the VA community, especially for those with limited English proficiency, remains limited. Past research has documented how male smoking and cessation efforts contribute to familial strain and communication challenges among VA families^19^. Additional research is needed to clarify how unique cultural and migratory experiences shape preventive health behaviors and maintenance of unhealthful practices among VA immigrants^20^.

This study is a part of our larger, community-partnered project (Project RAISE: Raising Awareness for Involuntary Exposure to Smoking) between BPSOS-Center for Community Advancement (BPSOS-CCA), a community organization that has been serving local VA communities since 2000 and the University of Southern California (USC). Using a sequential quantitative > qualitative explanatory mixed-methods design^21^, we assessed the prevalence of SHSe, rules around SHSe at home, and examined KAB about SHSe as a risk factor for breast cancer among VA women residing in Southern California across the lifespan. We hypothesized that prior and current SHSe among VA women would be higher than the national averages (during childhood: 40%; current exposure: 24.3%)^17,22^. We investigated individual – and family – level factors associated with prior and current SHSe to identify contextual variables that may be helpful to reduce SHSe and increase breast health awareness among the VA community. Following survey data collection, we qualitatively explored multilevel factors that contribute to SHSe among VA households with current smokers to contextualize our quantitative findings and probed health beliefs among the VA families with respect to breast cancer prevention and tobacco use/exposure.

## Methods

### Survey phase

#### Design

We conducted a community-partnered, mixed-methods, and cross-sectional study with VA women residing in Orange County, California, December 2024 – January 2026. We began with the survey phase followed by the interview phase. Participants completed a survey of 30–40 min (online via REDCap^23^ or paper-and-pencil) and received a $20 gift card upon completion. During a preliminary data check, we identified invalid response patterns with paper-and-pencil surveys, despite in-person assistance. Therefore, we opted to use the REDCap option exclusively, with necessary restrictions programmed. In-person assistance remained available for participants with limited technology skills who completed surveys at BPSOS-CCA.

#### Sample

From December 2024 to January 2026, we recruited 217 women who: 1) identified themselves as Vietnamese or VA; 2) resided in Southern California; and were 3) aged 18–65 years. Participants were recruited from door-to-door canvassing at multi-unit housing complexes, community outreach events, through social media, and from educational workshops held at BPSOS-CCA. We excluded illogical/invalid responses (n=35), resulting in an analytical sample of n=179.

#### Measures

##### Sociodemographic variables

Sociodemographic characteristics (age, country of birth, marital status, level of education) that could confound the associations of interest were assessed. We recoded marital status (married vs other) and education level (≤high school/GED vs some college and higher) to include in the multivariable regression models.

##### SHSe scale

SHSe was assessed for both retrospective and current exposure across multiple settings, including childhood exposure and current SHSe, yielding an index of cumulative SHSe^24^.

##### KAB regarding SHS exposure scale

Evaluates one’s understanding of SHS-related health risks and measures household smoking rules, the perceived impact of SHSe on children, and the perceived risk of nicotine products^24^.

##### Breast cancer screening beliefs questionnaire (BCSBQ)

This is a13-item instrument that evaluates cultural attitudes towards screening^25^, with three subscales: knowledge/perceptions of breast cancer (Cronbach’s α=0.82); barriers to mammography (α=0.75); and attitudes toward health check-ups (α=0.85). Higher scores indicate greater knowledge and positive attitudes, and fewer barriers.

##### Tobacco-related perceived breast cancer risk

We assessed perceptions on whether tobacco/combustible exposures would increase, decrease, or have no effect on breast cancer risk^25^.

#### Statistical analysis

We provided descriptive statistics for demographic characteristics, current and prior SHSe, indoor smoking household rules, KAB regarding tobacco exposure and breast cancer risks, preventive beliefs and practices. We compared the sample SHSe prevalence to the national average^17^ (24.3%) using one-sample chi-squared test and conducted bivariate analyses to examine the association between prior/current SHSe and contextual/demographic factors via chi-squared tests and t-tests. We also conducted multivariable regression analyses to investigate the association between preventive beliefs and practices and BCBSQ subscales, adjusting for age, country of birth, survey language, marital status and education level. All analyses were conducted in Stata v18.0^26^.

### Interview phase

#### Design

During July–November 2025, we conducted 60 min, one-on-one, semi-structured interviews with a new set of smoking status discordant family dyads to obtain smoker and non-smoker perspectives. Each family dyad member was interviewed in a separate interview room to facilitate discussions without being influenced by their family members. Interviews were recorded and conducted in-person at the BPSOS-CCA offices by 9 research assistants (1 interviewer and 1 notetaker assigned for each interview) who were trained and supervised by a team member with expertise in qualitative methods (RCC). Interviews conducted in Vietnamese (20 out of 22 interviews) were transcribed by 6 bilingual research assistants, re-checked by a different bilingual research assistant for accuracy, and later translated to English for analysis.

#### Sample

Interview participants were recruited through social media, community outreach events and workshops. The target range of sample size for the interview phase was 12–15 family dyads. Of 12 families recruited, one family member opted to stop during the interview. Thus, we collected interview data from 22 individual family members (11 non-smokers and 11 smokers; aged 25–65 years). Of the sample, 10 were husband-wife dyads, and one was a mother-son dyad. Each interview session was audio-recorded and transcribed verbatim. Our interview questions were guided by the Social Ecological Model Framework where multilevel contexts and domains relating to tobacco use and breast cancer beliefs and prevention, were explicitly probed^4,27,28^. The interim analysis of our survey data informed the interview questions to be further probed.

#### Data analysis

Vietnamese transcripts were translated into English then independently back-translated to Vietnamese by fully bilingual study members uninvolved in the initial translation to check for inconsistencies. Once the transcription was complete, study team members engaged in the Rapid Group Analysis Process (Rap-GAP)^29^. Rap-GAP is a novel, rigorous, yet efficient qualitative method that facilitates systematic analysis, while leveraging diverse perspectives of study team members. Study team members worked in pairs; each pair reviewed 4–8 transcripts, identify, compile and organize relevant quotes pertaining to a priori (deductive) and ground-up (inductive) themes, resulting in clusters of subthemes per larger themes as visual representation of relationships among the high-level themes. We denoted each analyzed quote by family ID (FID) and smoking status (NS: non-smoker; S: smoker). Subsequently, the study members participated in 1 h Zoom meetings (3+members required for each session), 3 times/week for 4 months, discussed direct quotes, and compiled and organized emerging themes through iterative discussions. We used a virtual, whiteboard tool, *Lucidspark*^30^, which allowed for real-time collaboration. When disagreement arose, interpretations were revisited and refined until group consensus was reached.

All study protocols, including the issues of research ethics and informed consent procedures, were approved by USC Institutional Review Board (UP- 24–00784).

## Results

### Survey data

#### Sample characteristics

The average age of study participants was 36.5 years (SD=15.2) (Table 1). Most of our participants were born in Vietnam (n=104; 58.1%), with the average duration of 13.2 years (SD=12.1) in the US; 48.6% were single, 35.8% were married; 93.3% had high school education/General Educational Development (GED) or higher; and 104 (58.1%) completed the survey in English. Most participants (74.3%) reported living with ≥3 people in the household, including themselves. Two participants (1.1%) were current smokers (both Vietnam-born).

**Table 1:** Sample sociodemographic characteristics of Vietnamese American women, prior and current secondhand smoke exposure prevalences, and knowledge, attitudes and behaviors about breast cancer risks and prevention, Southern California, December 2024–January 2026 (N=179)

Variables	n (%)
**Survey language**	
English	104 (58.1)
Vietnamese	75 (41.9)
**Age** (years), mean (SD)	36.5 (15.2)
**Country of birth**	
US	104 (58.1)
Vietnam	75 (41.9)
**Marital status**	
Single/never married	87 (48.6)
Married	64 (35.8)
Divorced/separated	16 (8.9)
In a domestic partnership	9 (5.0)
Widowed	3 (1.7)
**Education level**	
Lower than high school	12 (6.7)
High school diploma/GED	45 (25.1)
Some college/currently enrolled in college	35 (19.6)
College degree or higher	87 (48.6)
**Childhood SHSe at home**	93 (52.0)
US born	57 (54.8)
Vietnam born	36 (48.0)
**Childhood SHSe frequency**	
Rarely	8 (8.6)
Sometimes	62 (66.7)
Always	23 (24.7)
**Current SHSe**	77 (43.0)
At workplace	55 (30.7)
At home	44 (24.6)
In a car	38 (21.2)
**Explicit household rules prohibiting smoking indoors**	114 (63.7)
**KAB about tobacco smoke exposure and breast cancer risk**	
Increase	157.(87.7)
Decrease	0 (0.0)
Has no effect	7 (3.9)
Do not know	15 (8.4)
**KAB about e-cigarette vapor exposure and breast cancer risk**	
Increase	138.(77.5)
Decrease	2 (1.1)
Has no effect	7 (3.9)
Do not know	31 (17.4)
**KAB about cannabis smoke/vapor exposure and breast cancer risk**	
Increase	132 (73.7)
Decrease	1 (0.6)
Has no effect	14 (7.8)
Do not know	32 (17.9)
**KAB about SHSe and breast cancer risk**	
Increase	150 (85.2)
Decrease	1 (0.6)
Has no effect	7 (4.0)
Do not know	18 (10.2)
**Participants aged ≥40 years (N=66)**	
Ever done a mammogram	56 (84.9)
Done a mammogram in the past year	42 (63.6)

SHSe: Secondhand smoke exposure. KAB: knowledge, attitude and behavior.

#### Prior and current SHSe prevalence and rules about indoor SHSe at home

More than half (52.0%) reported SHSe inside their homes during childhood and adolescent years, 8.6% of whom reported rarely, 66.7% sometimes, and 24.7% reported always. Vietnam-born participants reported slightly higher levels of childhood SHSe (54.8% vs 48.0%) compared to US-born participants, but not statistically significant (χ^2^=0.81, p=0.37). Seventy-seven participants (43.0%) in our sample reported current SHSe from any sources; the most modal place for SHSe was workplace (30.7%), followed by inside the house (24.6%), and car (21.2%). Thus, the prevalence of SHSe in our sample exceeds the national prevalence of 24.3% (z=5.84, p<0.001). US-born participants reported higher levels of current SHSe compared to Vietnam-born participants (53.3% vs 35.6%, χ^2^=5.60, p=0.02). Despite the prevalent SHSe at home, only 63.7% of the participants indicated that they had explicit household rules about smoking indoors [i.e. strongly disagreeing with: ‘let visitors smoke in my home’ (70.4%) and ‘let family members smoke in my home’ (68.2%)].

#### KAB about tobacco exposure as a risk factor for breast cancer and child health

Most participants in our sample (64.8%) believed that tobacco smoking, vaping, cannabis smoking/vaping, and SHSe are associated with an increased risk of breast cancer, while 26.8% of the participants were unsure about their effects on breast cancer; 87.7% indicated that smoking increases the risk of breast cancer whereas 3.9% indicated smoking had no effect on the risk of breast cancer. Most participants (85.2%) reported SHSe increases risk of breast cancer and 4.0% reporting SHSe had no effect on risk of breast cancer, while 0.6% of the sample indicated that it decreases the risk of breast cancer. All participants but one (99.4%) believed SHSe to affect children’s health.

#### Preventive care and health beliefs

Overall, most participants (83.2%) valued the importance of discussing breast cancer screening with their healthcare providers. Among those recommended for breast cancer screening (aged ≥40 years; n=66), 56 (84.9%) indicated ever receiving screening and 42 (63.6%) indicated past-year mammograms (Table 1). For those who delayed screening (n=26), ‘I felt the medical care was not urgent’ was the most frequently cited reason (53.9%). Participants who had explicit household indoor smoking rules reported more proactive attitudes toward general health check-ups compared to participants without such rules [16.2 (SD=4.3) vs 13.6 (SD=4.2); t=3.86, p<0.001].

Furthermore, multivariable regression models showed that stronger beliefs that SHSe is harmful were positively associated with accurate knowledge and positive attitudes toward breast cancer treatment (β=0.90; 95% CI: 0.20–1.59), fewer barriers to mammography (β=0.99; 95% CI: 0.31–1.68), and proactive attitudes toward check-ups (β=0.98; 95% CI: 0.24–1.72), controlling for age, country of birth, survey language, marital status and education level. Current and past SHSe were not associated with BCSBQ subscale scores.

#### Prior SHSe and family smoking status

Participants living with a smoker during childhood (51.7% vs 25.4%, χ^2^=11.11, p=0.001) and those currently living with smokers (66.2% vs 29.8%, χ^2^=22.29, p<0.001) were more likely to report current SHSe compared to those who did not.

### Interview data with smoking-status discordant, family dyads

Guided by our survey data showing that 1 in 3 women lacked strict household rules about indoor smoking, we followed up with interviews to explore the cultural and familial contexts, given >85% of our survey sample believed that tobacco use, including SHSe, is associated with increased breast cancer risk. Our transcription and translation/backtranslation resulted in 277 pages of transcripts from over 24 h of interview audio. Iterative Rap-GAP discussions led to several themes reflecting culturally nuanced yet divergent views on male family members’ smoking behavior and family health. The quotes used in our Rap-GAP are organized by themes in Table 2.

**Table 2:** Rapid Group Analysis Process (Rap-GAP) quotes organized by themes of interview data: Vietnamese American family dyads in Southern California, July – November 2025 (N=22; N=11 non-smokers; N=11 smokers)

Themes	Insight	Quote
**Theme 1: Health beliefs (religious, spiritual, or cultural) leading participants to attribute health outcomes to fate/divine will, undermining motivations for preventive behaviors and cessation**	Health behaviors and related outcomes are dictated by fate or religious beliefs	*‘I found it very strange. She was very meticulous, she got proper health check-ups, she checked her eating carefully, and still got that ...When she was near passing, she kept mumbling, saying “God is punishing, God is punishing”. Maybe sometimes when people are in a critical moment, they don't know if they ever committed any sins, but they might feel maybe they did something wrong before and now are being punished, you know? Very pitiful. Felt sorry for her.’* (FID9, NS)
		*‘… so I'm not say like I'm one of that person that got choose. But it really depends on – it's something that miracle things in – in life ... So if you pray a lot maybe God will love you and he is not going to give you any cancer.’* (FID1, S)
	Beliefs leading to ambivalence about primary prevention of illness, placing greater emphasis on treatment after illness onset	*‘They don't really do preventative measure. And whenever I ask them about it, they'll be like, “Oh, everyone has their own like fate [sic] written. So if you got it, you treat it. If you, you cannot treat it and you die and that's it”.’* (FID1, NS)
		*‘Now, medicine is developed now, but illness is illness. We cannot tell from person to person ... My female cousin had breast cancer ... She had her breasts both removed. Both of them ... But the people around her don't smoke, her husband doesn't either, but she had – what was it – there’s a gene in the family.’* (FID3, NS)
	Faith-based coping among family members of smokers	*‘My husband smokes a lot, so honestly, talking about smoking makes me very sad and distressed. I truly wish – if I can be spiritual about it – for a divine power to awaken him and make him quit smoking because I see how harmful it is.’* (FID10, NS)
**Theme 2: A lack of availability of accurate health information contributing to uncertainty and diverging views about causal factors of breast cancer**	Various potential causal factors of breast cancer, cited by participants	*‘... I see around my life like everybody of like – living clean like healthy they always get cancer ... but I think it's about genes more.’* (FID1, S)
		*‘There's people who smoke their entire life and they don't get a single illness or ever sick a day in their life. And there's people who rarely smoke and still got some sort of cancer.’* (FID1, NS)
	Confusion about the importance of healthy, preventive behaviors	*‘[My] dad, who does smoke, doesn't have any sort of cancers. My mom, who doesn't smoke and who eats very healthy and nutritious, has two types of cancers. So I feel it's a hard sell [connecting smoking behaviors to cancer risk] whenever I try to talk to [smokers] about preventative measures.’* (FID1, NS)
	Distrust in the link between tobacco exposure and breast cancer	*‘And I know many people whose families don't smoke, and they still get it. So you can't blame it on smoking. If you smoke, the smoke goes into your lungs. I don't know if it goes to your breasts.’* (FID5, S)
	Cognitive bias among smokers leading to dismissing available data that demonstrate the harmful effects of smoking	*‘In reality, out in society there are many people who smoke but they still live to [be] eighty, ninety years old just fine. Not everyone who smokes dies early or everyone who smokes gets lung disease. There are many women who don’t even smoke, but they still get lung disease or cancer, or also die because of lung problems a lot.’* (FID11, S)
**Theme 3: Non-smoker females navigating familial tension surrounding cigarette smoking while maintaining relational harmony**	Cessation-related tensions in the households are ineffective in cessation or reduction	*‘My husband smokes about a pack a day, which is a lot. We’ve argued about it, but he has not reduced his smoking at all.’* (FID11, NS)
	Smoking bans initiated by non-smoker wives, resulting in inconsistent implementations	*‘I don't smoke inside the house. I don't smoke near my child. I can smoke in front of my wife, it's not a problem, but I don't want her to complain.’* (FID5, S)
		*‘He understands that it's for the good of the baby. But the funny thing is he will only agree to those rule if it involves the baby but not me … He would change his clothes and like brush his teeth if it was the baby – it is not me.’* (FID1, NS)
	Perceived rationale for the inconsistent smoking ban implementations	*‘No, I only ask that whenever he smokes, he goes to the back so it does not affect the children because I have already been affected a lot and don’t want the smoke to linger in the yard or inside the house. It’s in the back, a corner in the back so it won’t affect the house at all. Luckily our house is spacious and he is okay with going to that corner to smoke, he is okay with that.’* (FID11, NS)

FID: family ID; NS: non-smoker; S: smoker.

#### Theme 1: Health beliefs (religious/spiritual/cultural) led participants to attribute health outcomes to fate/divine will, undermining motivations for preventive behaviors and cessation

Both smoker and non-smoker participants shared their perspectives that health behaviors and/or related outcomes are dictated by fate or religious beliefs. Illness was attributed to God’s punishment for sins, suggesting that outcomes are predetermined despite preventative efforts. One non-smoker (FID9, NS) spoke of her friend who led a healthy lifestyle, receiving regular check-ups, died from cancer who perceived her illness as a faith-based, moral failure and inevitable, despite prevention (Table 2).

In contrast, a smoker participant (FID1, S) who identified himself as Catholic, expressed the belief that a strong faith in God would protect them against illness regardless of lifestyle or health behaviors, thus rendering preventive measures unnecessary as one can be ‘chosen’ through prayer and gain divine favor.

Some participants, regardless of smoking status, were ambivalent about engaging in primary prevention of illness, including health-promoting behaviors, as one’s destiny was believed to be determined by genetic makeup or by ‘fate’, thereby reducing preventive measures. This perspective was further reinforced in family interactions as seen in the quote from a non-smoking wife (FID1, NS), describing her in-laws' views on preventive health practices. She noted that her in-laws viewed illness as determined by fate, placing greater emphasis on treatment after illness onset over preventative measures. This perspective is echoed by another women (FID3, NS) who attributed her cousin’s cancer to genetic factors, reinforcing the belief that cancer is predetermined and up to fate rather than influenced by factors such as environmental exposures or modifiable behaviors, thereby highlighting the insignificance of prevention.

Faith-based coping appears to be exercised when non-smoker family members fail to enforce reductions in tobacco use and their own tobacco exposure, resulting in powerlessness as the family lacks available resources or knowledge (FID10, NS).

#### Theme 2: A lack of availability of accurate health information contributed to uncertainty and diverging views about causal factors of breast cancer

When probed their views on tobacco exposure and breast cancer, participants suggested various potential causal factors of breast cancer, ranging from genetic to lifestyles, other than tobacco (e.g. FID1, S and FID1, NS; Table 2). Participants further expressed preventive measures as ineffective and noted that smokers who never become ill, while others who never smoke still develop cancer and other illnesses, reinforcing the perception that cancer-related risks and chances of developing cancer are unpredictable and random. Regardless of smoking status, participants expressed confusion about the importance of preventive health behaviors, citing counterexamples from their own life in which healthy lifestyles do not prevent cancer, illustrated by a non-smoker who shared her attempts to encourage her husband to consider smoking-related cancer risks (FID1, NS). Smokers also expressed distrust in the link between tobacco exposure and breast cancer while acknowledging lung cancer risk (FID5, S).

Smokers tended to rationalize continued smoking, reflecting cognitive dissonance bias. They pointed to some smokers who live long healthy lives and speculated that smoking is not necessarily harmful to one’s health, rather than referring to available, well-established data that demonstrate the harmful effects of smoking on health. They also believed that non-smokers get ill, including cancer, therefore, not smoking may not necessarily be protective (FID11, S).

#### Theme 3: Non-smoker females navigate familial tension surrounding cigarette smoking while maintaining relational harmony

Divergent views about cigarette smoking and SHSe were expressed between sexes, which were confounded by the smoking status in our sample. Our female, non-smoker participants discussed varying levels of expressing disagreement with their smoker family members (mostly spouses), ranging from passively tolerating to annoyance to frustration. Female non-smoker participants often framed their behaviors and concerns as protecting and preserving household health while our male smoker participants perceived smoking behaviors as personal choice. Tensions and arguments within the household about smoking did not necessarily lead to successful cessation or reduction in use (FID11, NS; Table 2). Rather, they resulted in incomplete strategies for family health protection. Household indoor smoking bans were often initiated by the wives, strongly motivated by protecting children’s health (FID5, S). However, implementation was inconsistent, particularly regarding preventing SHSe for children versus non-smoking spouses (FID1, NS). Referring to prior SHSe cumulatively, one participant (FID11, NS) provided a rationale for this inconsistency that non-smoking spouses, had already been exposed to SHSe in previous years and protection from the harm may not be as efficacious compared to their offspring.

## Discussion

Our findings indicate that current SHSe and childhood SHSe in our sample significantly exceeded the national prevalences. The modal place for SHSe was at workplace, which is a shift from previous findings^16^. One in 4 in our sample is currently exposed to SHSe inside homes and cars. A higher proportion of US-born VA women reported SHSe than Vietnam-born VA women, which was unanticipated. Our US-born participants may have been exposed to more diverse public environments where SHSe is present (e.g. bars, coffee shops). Alternatively, US-born participants may have more exposure to public health warnings about SHSe, therefore, more aware of and/or more likely to detect SHSe.

Our data also showed the majority VA women endured the burden of SHSe inside their homes during childhood. Despite this cumulative and ongoing exposure as commonly modeled behavior, our qualitative data show that cigarette smoking is viewed as overwhelmingly male-dominant health behaviors in VA communities, also corroborated by the extremely low rate of current smoking in our survey data. The prevalent childhood SHSe among VA women, however, has implications for breast cancer given the burgeoning research that documents SHSe through premenarche up to the first childbirth (vs later in life) having greater adverse impacts on breast health^10,11^. Our findings from both survey and interview data indicated that participants were unsure and underinformed about the link between breast cancer and tobacco exposure, expressed by a quarter of our sample, consistent with prior research. In one recent study that assessed Vietnamese participants’ knowledge of breast cancer based on knowledge of symptoms, risk factors, and screening modalities, the level of knowledge of risk factors was the lowest^31^. Notably, neither smoking nor SHSe were included among the assessed risk factors. There are limited studies assessing VA communities’ awareness of the association between smoking and increased risk of developing various types of cancers. In one such study, respondents recognized increased risk for lung, mouth, throat, and esophageal cancer. However, recognition of the association between tobacco use and other types of cancer is significantly lower^32^. Markedly, breast cancer was not included in this study. The omission of SHSe in the assessment of breast cancer risk factor knowledge and the absence of breast cancer in smoking-related disease awareness suggests that gaps in participant knowledge may reflect, in part, how risk factors are defined and measured in the limited existing research. This underscores the need for effective public health education on tobacco exposure in relation to breast health among VA community.

Despite their beliefs that SHSe is harmful for children’s health and breast health, one third of our sample lacked explicit/strict rules about indoor smoking bans at home regarding visitors and family members. We further explored this through interview data and found that preserving familial harmony might be prioritized. Many interview participants expressed ambivalence and helplessness about recommending or supporting cessation, if such efforts were to cause familial tension. While cessation efforts tend to focus on the smoker, non-smoker family members encounter difficulties including their own physical, social, and emotional – often unmet – needs which can lead to contexts of powerlessness^33^.

Regarding preventive health behaviors, although VA women valued discussing screening with their healthcare providers and most reported having ever had a mammogram, one third of our sample who should do an annual mammogram indicated not following the recommendation. Our findings are consistent with a recent systematic review^34^ where consistently lower mammography screening rates were documented among VA women compared to the national rate. Language barriers and low familiarity with preventive practices have been associated with low mammography screening, which we also observed in our sample (e.g. ‘not urgent’). Similarly, for some male smokers, cessation was not their priority because they were healthy and they knew many other individuals who have smoked ‘all their lives’. Our Rap-GAP results provided insight that community hesitation may relate to the general belief that health, including getting sick and being cured from grave illness, is precarious; health has less to do with specific behaviors in which one engages. Rather, falling ill or being cured is unpredictable and beyond one’s control.

Another critical finding was that for cessation and/or SHSe reduction for VA community, the familial focal point would need to be on offspring, as most smokers have expressed concerns for SHSe impact on their children but less for their spouses. When non-smoker VA female family members bring up concerns and frustration about SHSe and even thirdhand smoke^35^, it was often considered a nuisance by their smoker male family members. For many of our smoker participants, the solution was temporary appeasement rather than complete cessation. This finding represents the great tension that requires thoughtful public health solutions where the group that is impacted the most can only express little in the matter to maintain familial harmony.

### Strengths and limitations

The study is grounded in community insights and opinions, as our study represents full collaboration and partnership between USC and BPSOS-CCA in all stages of the research project, spanning from research question formation, funding procurement, recruitment, data collection and analyses to dissemination. Our study instruments and protocols were also informed by community insights and input provided by our community advisory board members throughout the study period. This study also combined the strengths of both quantitative and qualitative research methods and leveraged perspectives from smokers and non-smokers among family dyads, exemplifying an equitable health research program where community opinions are maximally reflected in all stages of research from its conception to dissemination.

This study has several limitations. Given our sampling strategies, the sample is not representative; therefore, our findings may not be generalizable to all Vietnamese Americans residing in California. Our study employed a cross-sectional, mixed-methods study, which cannot establish causal relationships. Self-reported data are subject to misclassification and biases. The sample size might not have been adequately powered to detect meaningful associations of small magnitudes; omitted variables could have also contributed to potential residual confounding.

## Conclusions

Future community-based experimental studies are needed to design and test a community-led health campaign aimed at promoting cessation, breast cancer preventative care, and voluntary smoke-free policies in public spaces in Vietnamese American communities through family-centered communication strategies.

## References

[1] American Cancer Society (2026). Cancer facts & figures 2019; 2019. https://www.cancer.org/content/dam/cancer-org/research/cancer-facts-and-statistics/annual-cancer-facts-and-figures/2019/cancer-facts-and-figures-2019.pdf.

[2] Li CI, Malone KE, Daling JR (2003). Differences in breast cancer stage, treatment, and survival by race and ethnicity. Arch Intern Med.

[3] Thuc Nguyen TM, Dinh Le R, Nguyen CV (2023). Breast cancer molecular subtype and relationship with clinicopathological profiles among vietnamese women: a retrospective study. Pathol Res Pract.

[4] Nguyen-Truong CKY, Nguyen KQV, Nguyen TH (2018). Vietnamese American women’s beliefs and perceptions about breast cancer and breast cancer screening: a community-based participatory study. J Transcult Nurs.

[5] He Y, Si Y, Li X (2022). The relationship between tobacco and breast cancer incidence: a systematic review and meta-analysis of observational studies. Front Oncol.

[6] Kim AS, Ko HJ, Kwon JH, Lee JM (2018). Exposure to secondhand smoke and risk of cancer in never smokers: a meta-analysis of epidemiologic studies. Int J Environ Res Public Health.

[7] Lee PN, Hamling J (2006). Environmental tobacco smoke exposure and risk of breast cancer in nonsmoking women: a review with meta-analyses. Inhal Toxicol.

[8] Macacu A, Autier P, Boniol M, Boyle P (2015). Active and passive smoking and risk of breast cancer: a meta-analysis. Breast Cancer Res Treat.

[9] Callahan CL, Bonner MR, Nie J (2019). Active and secondhand smoke exposure throughout life and DNA methylation in breast tumors. Cancer Causes Control.

[10] Bonner MR, Nie J, Han D (2005). Secondhand smoke exposure in early life and the risk of breast cancer among never smokers (United States). Cancer Causes Control.

[11] Gram IT, Park SY, Wilkens LR (2025). Childhood exposure to Second-Hand Smoke (SHS) and risk of breast cancer in postmenopausal never smokers: the Multiethnic Cohort (MEC) study. Breast Cancer Res.

[12] Chan NL, Thompson B, Taylor VM (2007). Smoking prevalence, knowledge, and attitudes among a population of Vietnamese American men. Nicotine Tob Res.

[13] Kenny JD, Tsoh JY, Nguyen BH, Le K, Burke NJ (2020). Keeping each other accountable: social strategies for smoking cessation and healthy living in Vietnamese American men. Fam Community Health.

[14] Chae DH, Gavin AR, Takeuchi DT (2006). Smoking prevalence among asian americans: findings from the National Latino and Asian American Study (NLAAS). Public Health Rep.

[15] Martell BN, Garrett BE, Caraballo RS (2016). Disparities in adult cigarette smoking - United States, 2002-2005 and 2010-2013. MMWR Morb Mortal Wkly Rep.

[16] Ma GX, Tan Y, Fang CY, Toubbeh JI, Shive SE (2005). Knowledge, attitudes and behavior regarding secondhand smoke among Asian Americans. Prev Med.

[17] (2026). Secondhand smoke exposure. Cancer trends progress report. National Cancer Institute. Updated April, 2025. https://progressreport.cancer.gov/prevention/tobacco/smoke_exposure.

[18] (May 1, 2025). Facts about Vietnamese in the U.S. Pew Research Center. https://www.pewresearch.org/race-and-ethnicity/fact-sheet/asian-americans-vietnamese-in-the-u-s/.

[19] Petersen AB, Tsoh JY, Nguyen TT, McPhee SJ, Burke NJ (2016). Suffering in silence: impact of tobacco use on communication dynamics within Vietnamese and Chinese immigrant families. J Fam Nurs.

[20] Lee SY, Park CL (2018). Trauma exposure, posttraumatic stress, and preventive health behaviours: a systematic review. Health Psychol Rev.

[21] Palinkas LA (2014). Qualitative and mixed methods in mental health services and implementation research. J Clin Child Adolesc Psychol.

[22] Homa DM, Neff LJ, King BA (2015). Vital signs: disparities in nonsmokers’ exposure to secondhand smoke--united states, 1999-2012. MMWR Morb Mortal Wkly Rep.

[23] Harris PA, Taylor R, Minor BL (2019). The REDCap consortium: building an international community of software platform partners. J Biomed Inform.

[24] Vardavas C, Agaku I, Filippidis F (2017). The Secondhand Smoke Exposure Scale (SHSES): a hair nicotine validated tool for assessing exposure to secondhand smoke among elderly adults in primary care. Tob Prev Cessat.

[25] Kwok C, Endrawes G, Lee CF (2016). Breast cancer screening beliefs questionnaire: psychometric properties assessment of the Arabic version. Eur J Oncol Nurs.

[26] (2023). What’s new in Stata 18. StataCorp. https://www.stata.com/stata18/.

[27] Stokols D (1996). Translating social ecological theory into guidelines for community health promotion. Am J Health Promot.

[28] Scott N, Donato‐Hunt C, Crane M (2014). Knowledge, attitudes and beliefs about lung cancer in three culturally and linguistically diverse communities living in australia: a qualitative study. Health Prom J of Aust.

[29] Hsu C, Mogk J, Hansell L, Glass J, Allen C (2024). Rapid Group Analysis Process (Rap-GAP): a novel approach to expedite qualitative health research data analysis. Int J Qual Methods.

[30] (June 19, 2026). Transform your business with AI. Lucid. https://lucid.co.

[31] Ngan TT, Jenkins C, Minh HV, Donnelly M, O’Neill C (2022). Breast cancer screening practices among Vietnamese women and factors associated with clinical breast examination uptake. PLoS One.

[32] Ma GX, Tan Y, Feeley RM, Thomas P (2002). Perceived risks of certain types of cancer and heart disease among Asian American smokers and non-smokers. J Community Health.

[33] Monari EN, Booth R, Forchuk C, Csiernik R (2024). Experience of family members of relatives with substance use disorders: an integrative literature review. Creat Nurs.

[34] Nguyen AT, Duckworth ED, Li RA, Galiano RD (2026). Disparities in breast cancer screening, diagnosis, and outcomes among Vietnamese American women: a systematic review. J Surg Oncol.

[35] Vanzi V, Marti F, Cattaruzza MS (2023). Thirdhand smoke knowledge, beliefs and behaviors among parents and families: a systematic review. Healthcare (Basel).

